# Epigenetic and Immune Profile Characteristics in Sinonasal Undifferentiated Carcinoma

**DOI:** 10.1002/cam4.70413

**Published:** 2024-11-20

**Authors:** Hyang Joo Ryu, Chung Lee, Sun Och Yoon

**Affiliations:** ^1^ Department of Pathology Yonsei University College of Medicine, Severance Hospital Seoul Korea

**Keywords:** EZH2, head and neck cancer, polycomb repressive complexes, sinonasal undifferentiated carcinoma, spatial transcriptome analysis, tumor immunity

## Abstract

**Introduction:**

Sinonasal undifferentiated carcinoma (SNUC) is a rare and highly aggressive malignancy originating in the nasal cavity and paranasal sinuses. Its pathogenesis and immune characteristics remain poorly understood.

**Objectives:**

This study investigates the molecular aspects of SNUC, focusing on tumorigenesis and immunity.

**Methods:**

For this purpose, spatial transcriptome analysis was employed to compare the gene expression profiles of SNUC tumor cells with those of normal epithelial cells, as well as to compare tumor‐infiltrating immune cells with immune cells from normal, tumor‐free tissue areas. For validation, next‐generation sequencing tests and clinical sample studies were conducted.

**Results:**

Spatial transcriptome analysis revealed notable upregulation of EZH2 and the histone family gene such as H3C2 (H3‐clustered histone 2) in SNUC tumor cells. Additionally, gene set enrichment analysis identified significant activations in the histone deacetylase (HDAC) signaling pathway, histone acetyltransferase (HAT) pathway, polycomb repressive complex 2 (PRC2), and DNA methylation pathways. A notable decrease was observed in downregulated genes and pathways, including the mucin family of protein genes, the keratin protein gene, and the mucin glycosylation pathway. Next‐generation sequencing did not reveal specific genetic mutations within these pathways, although mutations such as *IDH2 R172S* were noted. Clinical SNUC tissues confirmed increased immunoexpression of EZH2 and PRC2 markers. Analysis of tumor immunity revealed a characteristic immune cell signature, with a notable predominance of naïve B cells, macrophages, CD8 memory T cells, and Tregs in the SNUC microenvironment, alongside the increased expression of LAG3 in tumor‐infiltrating immune cells.

**Conclusion:**

Our study suggests epigenetic mechanisms, particularly via EZH2, play a crucial role in SNUC carcinogenesis. Furthermore, distinctive immune cell profiles in SNUC point to potential immune‐related characteristics of this malignancy.

## Graphical abstract

Using spatial transcriptome analysis, evaluation of the epigenetic mechanisms in sinonasal undifferentiated carcinoma.

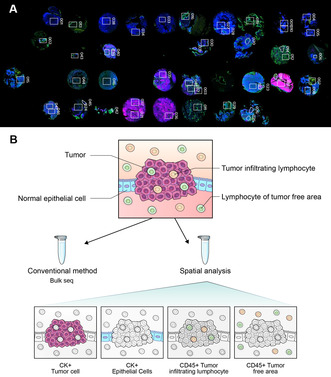

**Abbreviations**
AJCC: American Joint Committee on Cancer
DEG: differentially expressed genes
DSP: digital spatial profiling
FFPE: formalin‐fixed, paraffin‐embedded
GSEA: gene set enrichment analysis
HAT: histone acetyltransferase
HDAC: histone deacetylase
PRC: polycomb repressive complex
ROI: regions of interest
SNUC: sinonasal undifferentiated carcinoma
TIME: tumor immune microenvironment
TMA: tissue microarray

## 1 Introduction

Sinonasal undifferentiated carcinoma (SNUC) is a malignant epithelial neoplasm originating in the nasal cavity and paranasal sinuses. The diagnosis of SNUC is established when cytokeratin‐positive tumor cells show no histological or immunohistochemical evidence of differentiation into squamous, glandular, or neuroendocrine cells. As such, SNUC is often diagnosed through a process of exclusion [1]. Accounting for about 3%–5% of sinonasal carcinomas, SNUC is relatively rare. It is recognized as a highly aggressive cancer, characterized by a poor prognosis, with an estimated 5‐year survival rate of around 35%. Upon diagnosis, SNUC often presents with large, rapidly expanding masses that affect multiple areas within the sinonasal tract and often invade adjacent structures, including the orbital cavity, skull base, and brain [1, 2, 3].

SNUC, first identified in 1986 [3], still has an inadequately understood pathogenesis. Research into the carcinogenesis of SNUC points to a correlation between tumor development and recurrent *IDH2* R172 mutations [4, 5, 6]. These mutations are known to induce H3 lysine methylation and DNA hypermethylation [7]. Additionally, alterations in the SWI/SNF chromatin remodeling complex [8], known for its tumor‐suppressing roles [9], and the *PGAP3‐SRPK1* fusion [8], whose specific function in oncogenesis remains unclear but is implicated in SNUC, have been observed. These limited results, however, do not fully elucidate the pathogenesis of tumor development in SNUC.

In terms of tumor immunity in SNUC, there has been a documented case where a patient with SNUC exhibited a complete response following anti‐PD‐1 immunotherapy [10]. However, research on the impact of this therapy on SNUC's immune response is still limited [10, 11]. Although the concept of the tumor immune microenvironment (TIME) has gained recent attention in the context of tumor development, [12, 13] studies focusing on the tumor immunity of SNUC are scarce, leaving the role of TIME in this particular cancer not well understood.

The rarity and limited understanding of SNUC's pathophysiology likely contribute to the absence of a standardized treatment regimen for this aggressive tumor [14, 15]. Research on SNUC faces technical obstacles, such as challenges in accessing the lesion site and procuring sufficient tissue samples. Additionally, samples are sometimes subjected to a decalcification process, which can further complicate research efforts. These factors not only make it difficult to obtain a significant number of viable SNUC tumor cells but also hinder the development of cell lines or animal models for experimental research.

In our study, we concentrated on the carcinogenesis and tumor immunity aspects of SNUC. To circumvent the technical challenges, we performed spatial analysis of the tumor transcriptome directly within SNUC tissues. Our approach involved two key comparisons: First, we contrasted the gene expression profiles of SNUC tumor cells with those of normal epithelial cells in the sinonasal tract to gain insights into the carcinogenic processes of SNUC. Second, we compared the transcript levels of tumor‐infiltrating immune cells to those in immune cells from normal, tumor‐free tissue areas, aiming to explore the potential influence of the TIME in SNUC. Validation of our findings was conducted using clinical tissue samples. The primary goal of this research was to uncover molecular characteristics that are significantly involved in SNUC's development, with a particular focus on its carcinogenesis and tumor immunity.

## 2 Materials and Methods

### 2.1 Patients, Samples, and Clinical Data

From the database of Severance Hospital Cancer Registry Data in Seoul, Korea, approximately 30 cases that underwent either surgical excision or biopsy were reviewed. Each case was pathologically confirmed to be SNUC, sampled from the primary site of the sinonasal tract. Archived formalin‐fixed, paraffin‐embedded (FFPE) tissue samples were obtained. However, samples that had undergone decalcification, chemotherapy, or had a limited number of tumor cells were excluded. Ultimately, 23 cases were included in the study. To select the most representative sections, samples were mounted on slides, stained with hematoxylin and eosin (H&E), and reviewed by two pathologists (H.J.R. and S.O.Y.). All methods and experimental protocols involving human tissues were conducted in accordance with relevant guidelines and regulations, and the need for informed consent to participate was waived by the Institutional Review Board (IRB) at Institutional Review Board of Severance Hospital, Yonsei University Health System (IRB no. 4‐2022‐0466).

### 2.2 Immunohistochemical Staining, In Situ Hybridization, and Microscopic Analysis

The representative slides were selected for immunohistochemical analysis. Immunohistochemistry was performed on 4 μm thick TMA sections using a Ventana BenchMark XT autostainer (Ventana Medical Systems, Tucson, AZ), following the manufacturer's instructions. The primary antibodies used included CK(AE1/AE3) (1:50, clones AE1/AE3; Dako), p40 (RTU, polyclonal; Biocare), p63 (1:50, clone DAK‐p63; Dako), CK5/6 (1:50, clone D5/16 B4; Dako), p16 (RTU; Ventana), CK7 (1:100, clone OV‐TL 12–30; Dako), NUT (1:50, clone C52B1; Cell Signaling), Synaptophysin (1:50, clone DAK‐SYNAP; Dako), Chromogranin A (1:100, clone DAK‐A3; Dako), INI‐1(SMARCB1) (1:200, clone BAF47; BD), BRG1(SMARCA4) (1:200, clone EPNCIR111A; Abcam), EZH2 (1:20, clone ZMD.309; Invitrogen), BMI1 (1:100, clone 1.T.21; Abcam), SUZ12 (1:50, clone SUZ220A; Abcam), EED (1:100, clone 163C; Abcam), and H3K27me3 (1:200, clone C36B11; Abcam). In situ hybridization (ISH) was performed using the Ventana EBER ISH iView Blue Plus Kit (Ventana). Each marker was subjected to either semiquantitative or qualitative analysis by expert pathologists. The analysis criteria were based on either a cutoff value (generally, more than 10% for positive expression) or an H‐score (on a scale from 0 to 300, calculated as the proportion of positive tumor cells, ranging from 0% to 100%, multiplied by the nuclear expression intensity, scored from 0 to 3).

### 2.3 Multiplex Digital Spatial Profiling (DSP) Analysis

The NanoString GeoMx DSP assay (NanoString Technologies, Seattle, WA, USA) was performed in 23 cases of SNUC. Under microscopy, tumor and normal tissue areas were identified on H&E‐stained slides for each case. One to two tumor cores and one normal core were extracted from each FFPE tissue block using a 2 mm punch. These cores were then assembled into a tissue microarray (TMA). The TMA comprised a total of 38 cores, including 27 tumor cores and 11 normal cores.

**FIGURE 1:**
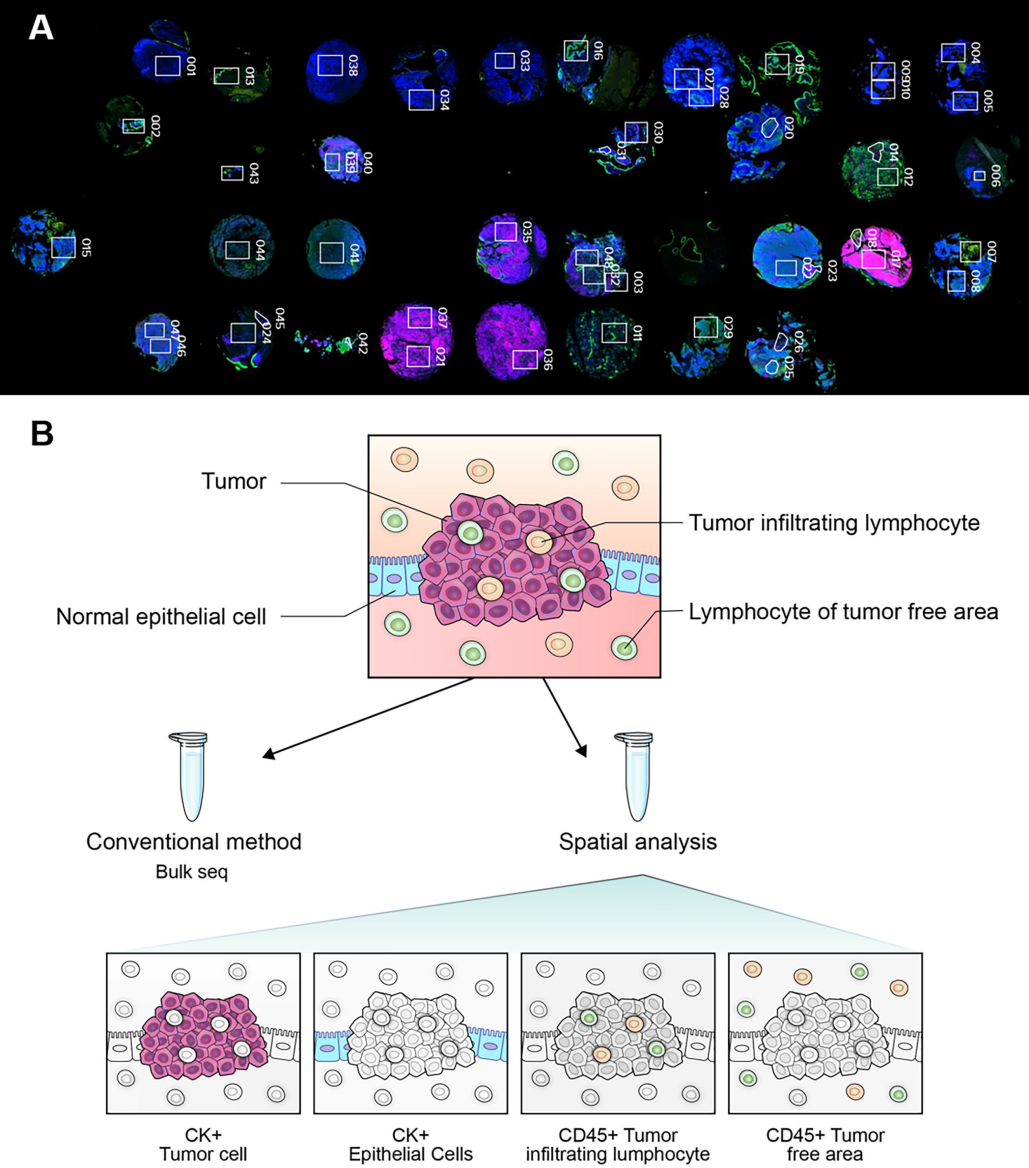
Representative images and schematic diagram for DSP analysis. Panel (A) displays immunofluorescence images utilized in digital spatial profiling (DSP) analysis, emphasizing the selected regions of interest (ROIs). Panel (B) presents a schematic diagram illustrating the concept of cell segmentation in DSP.

For differential visualization of three primary cell types—carcinoma cells, lymphocytes, and stromal fibroblastic cells—pan‐cytokeratin, CD45, and SMA were stained using an immunofluorescence assay on the TMA slides. One to three geometric regions of interest (ROIs) were targeted in each TMA core for GeoMx multiplex DSP analysis, resulting in a selection of 48 ROIs. These ROIs included 37 from tumor tissue areas and 11 from normal tissue areas, as illustrated in Figures 1 and S1.

To segment the epithelial cells (Figure 1B), which comprised both tumor cells and normal epithelial cells, cytokeratin (CK)‐positive signals were evaluated and compared with H&E‐stained microscopic morphology. This led to the selection of 36 ROIs, with 28 from the tumor epithelial cell area and 8 from the normal epithelial area. For immune cell segmentation (Figure 1B), CD45‐positive signals were assessed alongside H&E‐stained morphology, resulting in the selection of 10 ROIs—7 from tumor‐infiltrating immune cell areas and 3 from normal areas. Selection of each region of interest (ROI) was conducted by expert pathologists. The details of each segmented area, including CK‐positive tumor cells, normal epithelial cells, and CD45‐positive immune cells within tumor or normal tissue regions, are summarized in Figure S1.

We used a validated panel, the GeoMx Whole Transcriptome Atlas (Human RNA) with NGS readout from NanoString Technologies, which encompasses over 1800 RNA targets. All ROIs were subjected to quality control testing using the Q3 normalization method, and all successfully passed this QC test. The specifics are detailed in Table S1.

### 2.4 Next‐Generation Sequencing (NGS) Analysis

Targeted deep sequencing analyses, including genome analyses, was performed at Macrogen (Seoul, Korea), using FFPE DNA from SNUC tissues. DNA extraction followed the QIAamp DNA FFPE Tissue Kit protocol (Qiagen). Quality checks involved electrophoresis and the Qubit dsDNA HS Assay Kit (Life Technologies). DNA was fragmented to 180–200 bp using Adaptive Focused Acoustics (Covaris). Libraries, prepared with the SureSelect XT protocol (Agilent Technologies) and Axen Cancer Master panel (Macrogen, Korea), underwent quality assessment with a 2100 Bioanalyzer (Agilent), targeting 200–400 bp product size. Sequencing was performed on a NextSeq500 system, achieving approximately 2000× coverage with paired‐end reads (2 × 150 bp). The specific gene regions are outlined in Table S2. Reads, aligned to GRCh37/hg19 using BWA‐MEM, excluded poorly mapped and duplicated reads and recalibrated base quality. Somatic mutations, including SNVs and INDELs, were identified using MuTect2, filtering out false‐positives and variants below 2% variant allele frequency (VAF) and 100× depth. Germline variants were excluded based on their minor allele frequency (MAF) in ExAC_EAS or the Macrogen Korean Population Database. Variants were annotated with SnpEff and SnpSift v4.3i and dbNSFP v2.9.3. Microsatellite instability (MSI) was assessed using mSINGS, and tumor mutational burden (TMB) was reported as mutations per megabase of passed missense mutations.

### 2.5 Estimated Immune and Stromal Cell Profile Using SpatialDecon Library With SafeTME Matrix

To discern immune and stromal cell profiles in SNUC samples, SpatialDecon analysis was conducted using the SafeTME cell profile matrix derived from 75th percentile normalized RNA expression data. The estimated immune cell types for each region of interest (ROI) were illustrated in a heatmap. The methodology is detailed in previous research [16].

### 2.6 Statistical Analysis

Statistical significance between two variables was assessed using Chi‐squared test, Fisher's exact test, Student's *t* test, and Wilcoxon signed‐rank test. *p*‐values less than 0.05 were deemed significant, with all reported *p*‐values being two‐sided. Data analysis was performed using R software (version 4.3.1). Protein expression from three independent Western blots was quantified using Image J Software, normalized against the corresponding beta‐actin signals in the samples.

## 3 Results

### 3.1 Clinical and Pathological Features

**FIGURE 2:**
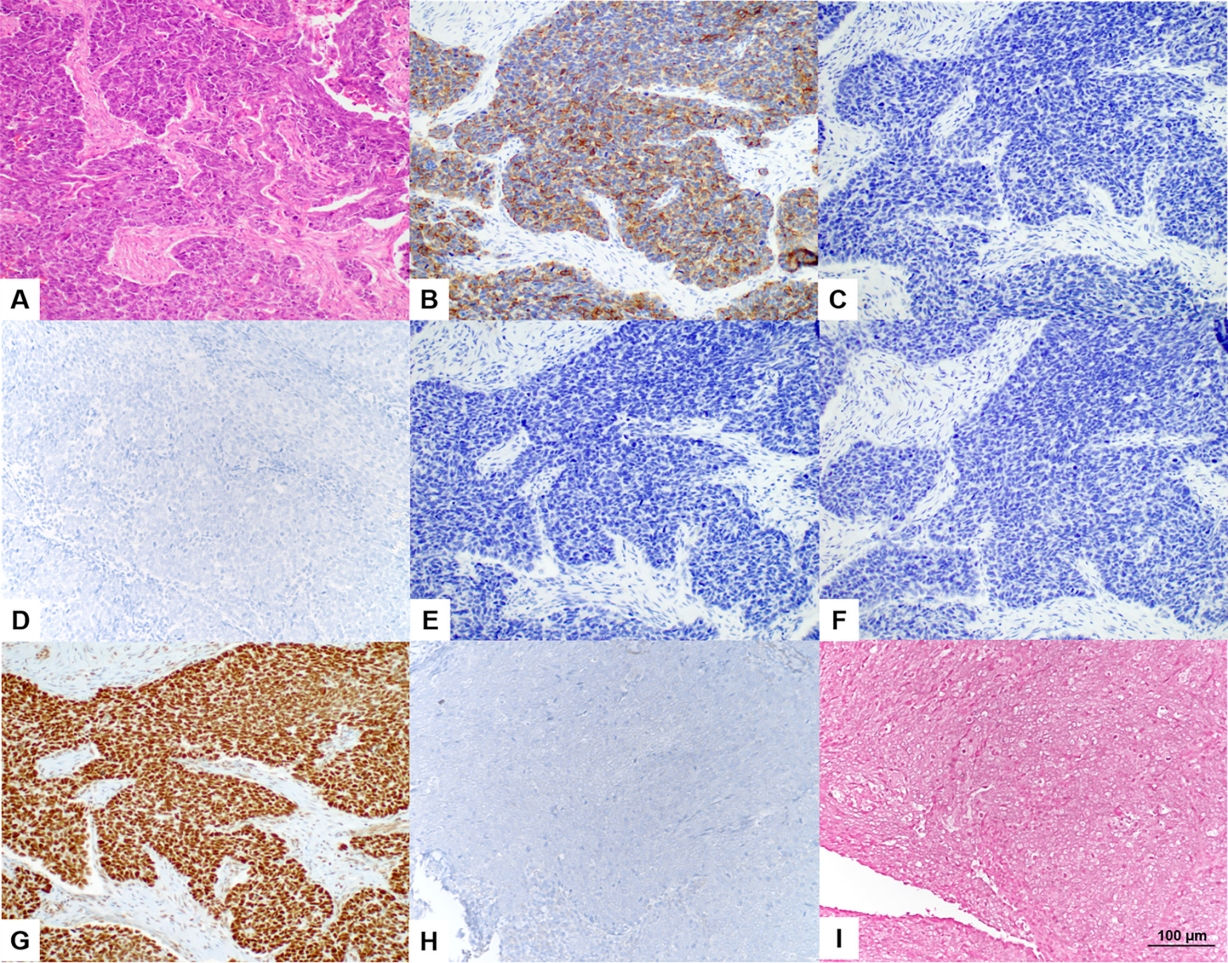
Representative images of sinonasal undifferentiated carcinoma (SNUC) in hematoxylin and eosin (H&E) staining, immunohistochemical staining, and in situ hybridization. Panel A shows diffuse infiltration of undifferentiated carcinoma cells with numerous mitotic features, lacking specific histologic patterns in an HE stain image. The tumor cells exhibit focal positivity for CK(AE1/AE3) (B) and are negative for p40 (C), CK7 (D), synaptophysin (E), and chromogranin A (F). All cases retained the expression of INI1 (G). Additionally, all cases were negative for NUT (H), and the majority of tumor cells in each case were negative for EBER in situ hybridization (I). All images were captured at 200x magnification.

The clinical characteristics of the study cohort are summarized in Table 1. It comprised 17 (74%) men and six (26%) women, with a median age of 54 years. More than half of cases (13 out of 23, 57%) were diagnosed at the advanced tumor stage of American Joint Committee on Cancer (AJCC) III–IV. The median disease‐specific survival period was 60.4 months. Distant metastasis was observed in 6 (26%) of the 23 patients. The histological diagnosis followed the WHO tumor classification's 5th edition definition of SNUC [1]; SNUC shows no specific differentiation into squamous, glandular, or neuroendocrine lineage in histology and immunophenotype. Histologically, the tumor cells exhibited large, monotonous, undifferentiated morphology with prominent nucleoli and scant cytoplasm. In most cases (91.3%, 21 out of 23), variable degrees of tumor necrosis were present. All cases showed cytokeratin expression with varying intensity and distribution. Other markers assessed did not significantly indicate squamous differentiation (p40 and p63), glandular differentiation (CK7), neuroendocrine differentiation (synaptophysin and chromogranin), NUT expression, human papillomavirus (HPV) association (p16), Epstein–Barr virus infection (EBER), or deficiencies in INI1 (SMARCB1) and BRG1 (SMARCA4). Only one case showed a loss of BRG1 (SMARCA4). Nonetheless, NGS analysis in this case revealed no *SMARCA4* genetic mutations, and it was included in the study. Representative images are presented in Figure 2.

**TABLE 1:** Clinical characteristics of 23 patients with sinonasal undifferentiated carcinoma.

Characteristics	Number (%)
Age, median (range), years	54 (29–86)
Sex	
Male	17 (73.9)
Female	6 (26.1)
Initial stage, according to AJCC stage	
I	1 (4.3)
IIB	1 (4.3)
III	3 (13.1)
IV	10 (43.5)
Unknown	8 (34.8)
Metastasis	
Absent	17 (73.9)
Present	6 (26.1)
Liver	1
Bone	3
Lung	1
Distant lymph node	1
Overall disease‐specific survival, median	60.4 months

Abbreviation: AJCC, American Joint Committee on Cancer.

### 3.2 Spatial Transcriptome Features of Carcinogenesis Among CK‐Positive Cell Populations

**FIGURE 3:**
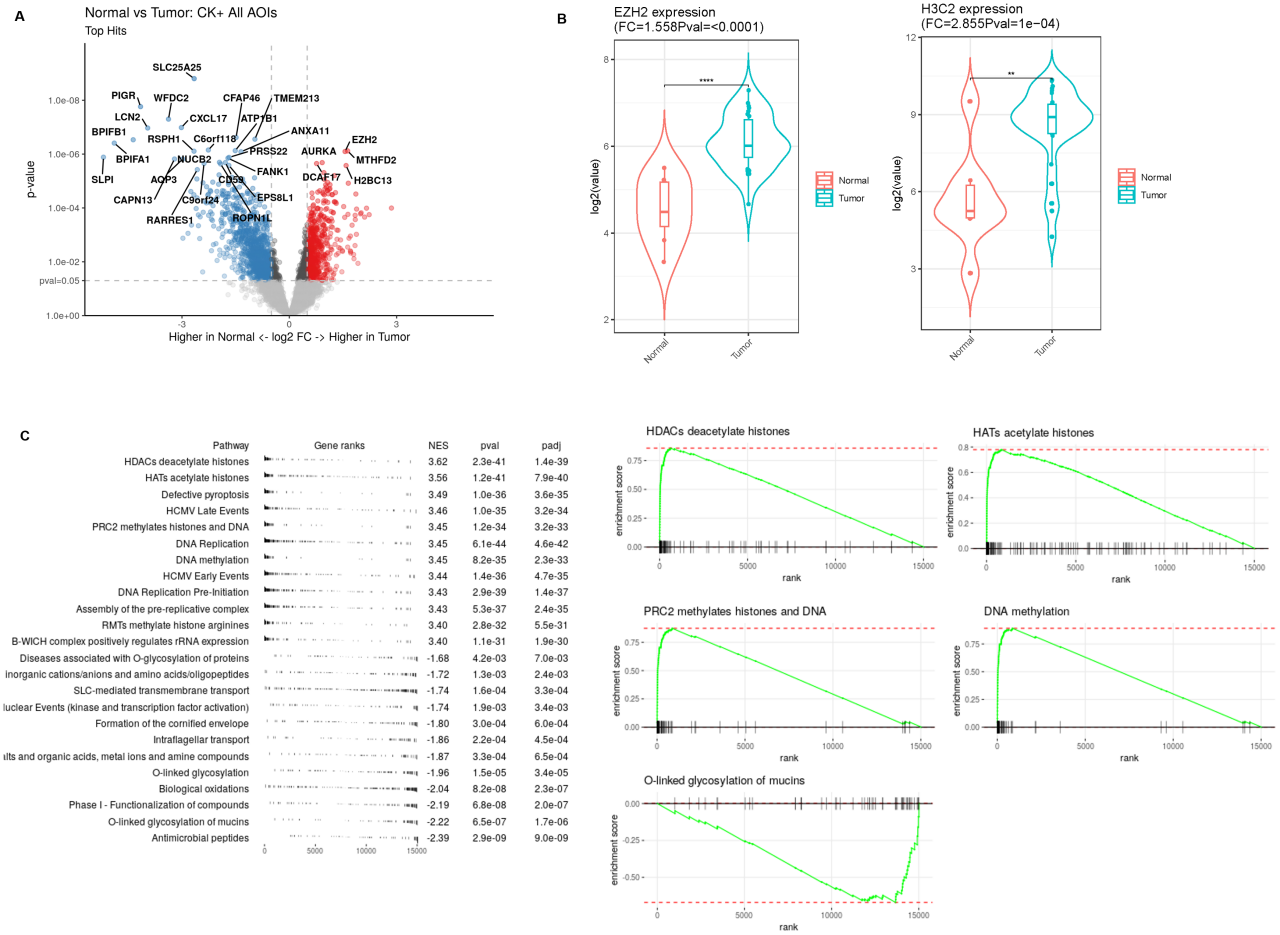
(A) Volcano plot illustrating differential gene expression. This plot highlights the significant upregulation of EZH2 in carcinoma cells compared to normal epithelial cells, which are cytokeratin‐positive. Fold change and significance levels (*p*‐values) are calculated based on log2‐transformed Q3‐normalized counts. (B) Wilcoxon signed‐rank test images displaying gene expression status related to carcinogenesis. Carcinoma cells in SNUC show significant upregulation of EZH2, as well as histone family genes like H3C2 (H3‐clustered histone 2), when compared to normal epithelial cells of the sinonasal tract. The gene set enrichment analysis (GSEA) reveals significant activations in pathways including histone deacetylase (HDAC), histone acetyltransferase (HAT), polycomb repressive complex 2 (PRC2), and DNA methylation pathways (C). In terms of downregulated genes and pathways in SNUC, a marked decrease was observed in the mucin glycosylation pathway (C).

The gene expression status of tumor cells was compared with that of normal epithelial cells using GeoMx multiplex DSP analysis, followed by differentially expressed gene (DEG) analysis. The overall details are summarized in Table S3, with notable differences illustrated in Figure 3. Among the genes identified as significantly upregulated or downregulated in SNUC, EZH2 and the histone family gene, such as H3C2 (H3 clustered histone 2), were notably upregulated in SNUC tumor cells (fold change > 1.5 times, *p* < 0.05). This is illustrated in Figure 3A,B, and further detailed in Tables S3 and S4, in comparison to normal epithelial cells. In the gene set enrichment analysis (GSEA), significant activations were observed in the histone deacetylase (HDAC) signaling pathway, histone acetyltransferase (HAT) pathway, polycomb repressive complex 2 (PRC2), and DNA methylation pathways, as detailed in Figure 3C. Regarding downregulated genes and pathways in SNUC, a notable decrease was observed in the mucin family of protein genes (MUC5B, MUC4, MUC1, and MUC5AC) and the keratin protein gene (KRT7) (fold change < −1.5 times, *p* < 0.05), as well as in the mucin glycosylation pathway, which are detailed in Figure 3C, and Tables S3 and S5.

### 3.3 Spatial Transcriptome Features of Tumor Immunity Among CD45‐Positive Cell Populations

The gene expression of each group of CD45‐positive immune cells in tumor areas (Figure S2A) and tumor‐free normal tissue areas (Figure S2B) was analyzed using GeoMx multiplex DSP analysis.

**FIGURE 4:**
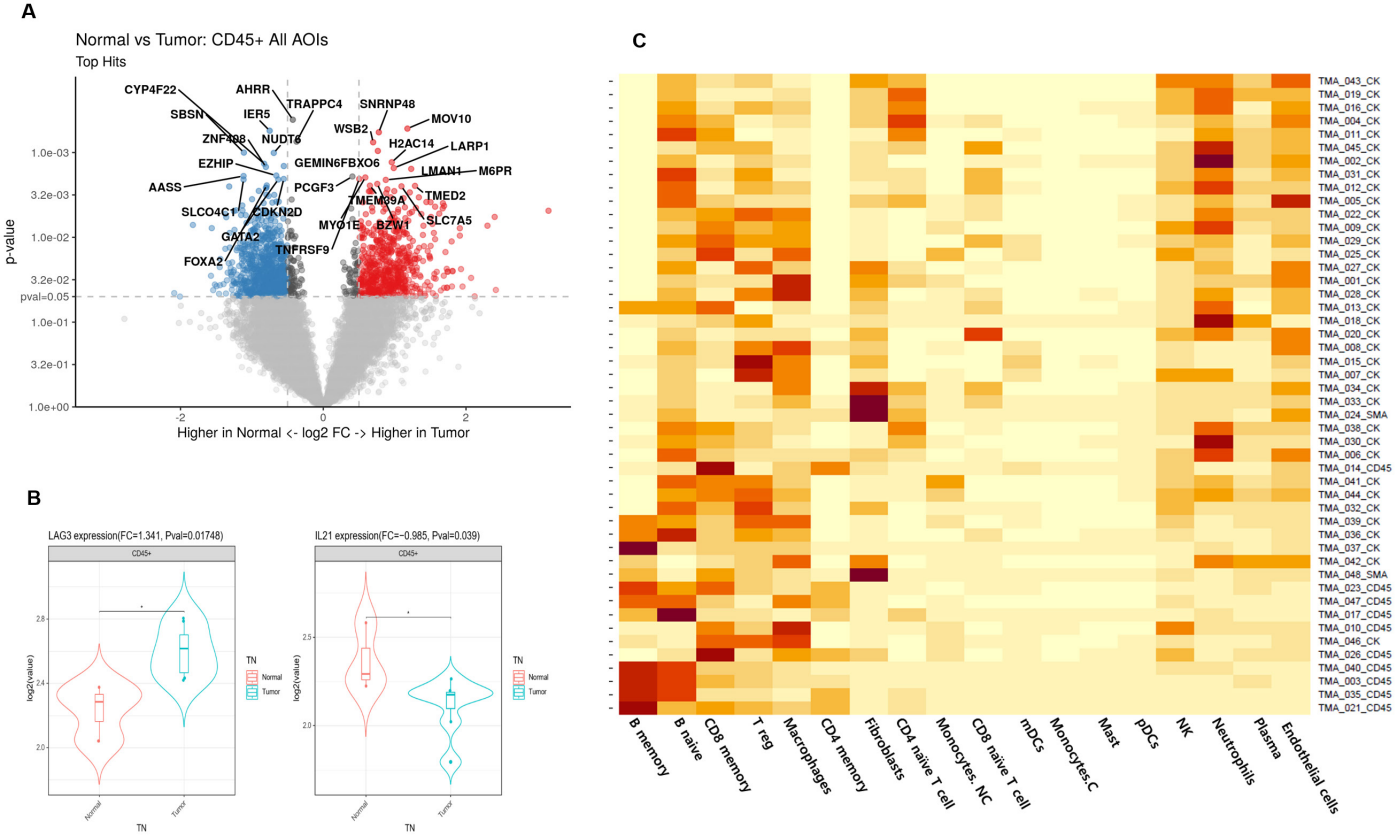
Volcano plot illustrates differential gene expression in CD45‐positive immune cells of the tumor area compared to those in the tumor‐free area. Some genes are identified as showing significant upregulation or downregulation, as detailed in the section corresponding to results with a *p* < 0.05 and a fold change > 1.5 or < −1.5. (A) A Wilcoxon signed‐rank test demonstrated significant upregulation of LAG3 and downregulation of IL21. (B) Estimation of the immune and stromal cell profile was conducted using the SpatialDecon library SafeTME matrix. In a total of 48 regions of interest (ROIs), increased signatures of naive B cells, macrophages, CD8 memory T cells, and Tregs were frequently observed (C).

When comparing tumor‐infiltrating immune cells with those in tumor‐free normal tissue areas, certain genes showed significant upregulation or downregulation (*p* < 0.05 and fold change > 1.5 or < −1.5). These details are summarized in Figure 4A and Table S6. While LTF and TUBA3D were identified as significantly downregulated genes, their roles in cancers remain unclear. Analysis of genes related to TIME [12, 13] revealed no significant expression changes, except for the relative overexpression of LAG3 in tumor‐infiltrating immune cells and a relative decrease in IL21 expression (Figure 4B). Detailed results from the Wilcoxon signed‐rank test for individual genes related to TIME are available in Figure S3.

In the estimated immune and stromal cell profile, using the SpatialDecon library with the SafeTME matrix and displaying the fold change of immune cell profiles, an increase in signatures of naïve B cells, macrophages, CD8 memory T cells, and Tregs was frequently observed across all 48 ROIs (Figure 4C).

### 3.4 Investigating Genetic Alterations

To figure out the causes of activated chromatin remodeling and histone modification pathways, NGS was employed to detect any relevant genetic alterations. The NGS study was conducted on eight cases. The remaining 15 cases were ineligible for NGS testing due to quality control (QC) failures, leaving only 8 that passed QC and were successfully tested. All eight cases were classified as having a low TMB and exhibited no MSI status.

No mutations were observed in the histone modification pathways of HDACs and HATs, or in the PRC2 chromatin remodeling pathway (involving EZH2, SUZ12, and EED). Alterations in the BMI1 gene, a component of PRC 1, were not confirmed, as it was not included in the NGS panel.

**FIGURE 5:**
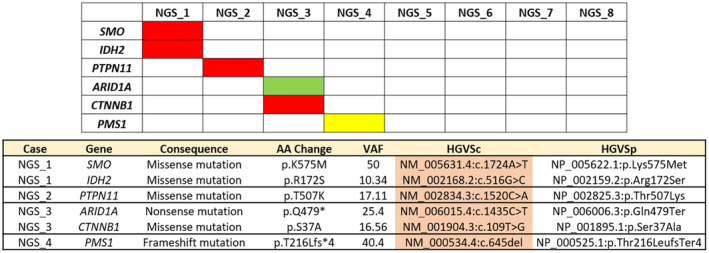
Detailed variant of clinical significance (VCS) information for the eight cases. Missense mutations are indicated in red, nonsense mutations in green, and frameshift mutations in yellow.

Among the eight cases, mutations in the *IDH2 R172S*, *SMO*, *PTPN11*, *CTNNB1*, *ARID1A*, and *PMS1* genes were identified as variants of clinical significance (VCS) in four cases. The details of these detected VCS are summarized in Figure 5.

### 3.5 Validation Analysis of PRC Activation in Clinical Samples

**FIGURE 6:**
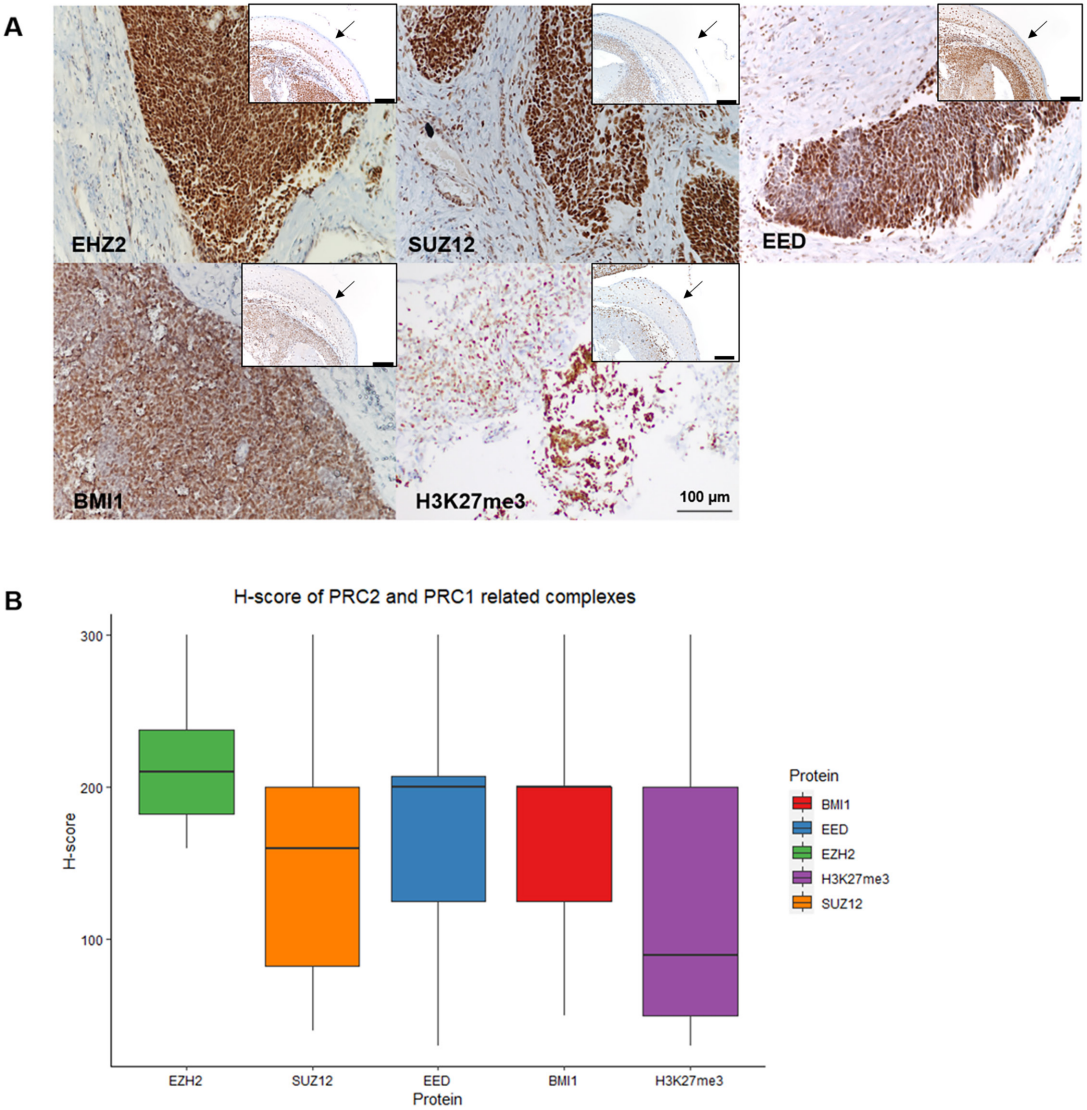
(A) Representative immunohistochemical stain image of PRC1/2‐related expression. This image shows EZH2, BMI1, EED, SUZ12, and H3K27me3. The arrow in the inset image is pointing to the expression of normal squamous epithelium. All images were captured at 200× magnification. (B) Boxplot image of H‐score results for PRC1/2‐related complex expression. The boxplot illustrates that EZH2 expression is notably high, while the expressions of other proteins display a relatively broad range.

In the clinical tissue samples of SNUC, the immunohistochemical expression of EZH2, SUZ12, EED, BMI1, and H3K27me3 was evaluated. The expression status, as represented in Figure 6, revealed that EZH2 was highly expressed in most cases, with a median H‐score of 210 (ranging from 160 to 300). Similarly, increased expression was observed for EED (with a median of 200, ranging from 30 to 300), BMI1 (median of 200, range 50–300), and SUZ12 (median of 160, range 40–300). The expression of H3K27me3 varied widely, with a median score of 90 and a range from 30 to 300.

## 4 Discussion

This study aimed to figure out the molecular characteristics of SNUC, from the perspectives of tumorigenesis and tumor immunity. Our SNUC cohort showed clinical and pathological features typical of aggressive carcinoma. Over half of the cases presented with an advanced tumor stage at diagnosis and frequently showed distant metastasis.

Spatial transcriptome analysis offers a significant advantage by enhancing the purity of the cells studied compared to traditional bulk sequencing methods. Bulk sequencing often struggles with a mixture of various cell types, making the isolation of specific cells of interest challenging. In contrast, recent advancements in spatial transcriptome analysis have enabled us to accurately categorize and examine four distinct cell types: Tumor cells, normal epithelial cells, tumor‐infiltrating immune cells, and immune cells in tumor‐free areas. This approach has facilitated a more comprehensive and detailed investigation (Figure 1).

Our spatial transcriptome analyses revealed activated pathways in the PRC2, the HDAC signaling pathway, the HAT pathway, and the DNA methylation pathway, including the upregulation of EZH2. These results suggest that epigenetic regulation, such as chromatin remodeling, histone modification, and DNA methylation pathways, may play a dominant role in SNUC carcinogenesis. In terms of downregulated genes and pathways in SNUC, we observed a decrease in mucin proteins (MUC5B, MUC4, MUC1, and MUC5AC) and the keratin protein (KRT7), along with a reduction in the mucin glycosylation pathway. These findings might indicate an alteration in the typical cellular characteristics of the normal mucosal epithelium in the sinonasal tract as the tumor progresses to undifferentiated carcinoma [17, 18, 19].

In our NGS analyses aimed at identifying the underlying causes for the activation of epigenetic regulation, including chromatin remodeling and histone modification pathways, we found no genetic mutations associated with these pathways. Specifically, mutations in HDACs, HATs, DNMTs (DNA methyltransferases), or the PRC chromatin remodeling pathway (EZH2, SUZ12, and EED of PRC2) were not detected. Our NGS results in SNUC suggest that the activation of chromatin remodeling and histone modification pathways might occur through mechanisms other than genetic alterations. Notably, actionable or druggable gene mutations in EZH2, SUZ12, or EED are mostly observed in hematologic malignancies. Conversely, actionable mutations in HDAC or HAT have not been well defined in both solid and nonsolid cancers [20, 21, 22]. However, our study has a limitation: The NGS analysis was conducted on only 8 of the 23 cases due to a lack of tissue samples. Therefore, we cannot conclusively decide the role of genetic mutations in inducing alterations in chromatin remodeling and histone modification pathways in SNUC. Further studies with more tissue samples are needed to explore this aspect.

Interestingly, we identified mutations in the *IDH2 R172S*, *SMO*, *PTPN11*, *CTNNB1*, *ARID1A*, and *PMS1* genes as variants of clinical significance (VCS) in four cases. *IDH2* hotspot mutations have recently been identified in SNUC, with the prevalence ranging from 33% to 85%. Notably, the *IDH2* R172S mutation is the most commonly observed among these [4, 5, 23, 24].

*IDH* mutations are widely recognized for their alleged roles in inducing DNA hypermethylation in various malignancies, including glioma, myeloid leukemia, cholangiocarcinoma, and chondrosarcoma [25]. In SNUC, the presence of *IDH2* mutation is associated with a hypermethylation phenotype. Studies focusing on DNA methylation profiling suggest that IDH‐mutant sinonasal malignancies represent a distinct category, differing from tumor types without IDH mutation [6].

SWI/SNF complex‐deficient sinonasal carcinoma has been recently recognized as a distinct entity in the WHO classification [1]. Similar to SNUC, this tumor in the sinonasal tract is characterized as a poorly differentiated to undifferentiated carcinoma. It is defined by the loss of one of the SWI/SNF complex subunits (either SMARCB1/INI1 or SMARCA4/BRG1), leading to its categorization as a specific entity. The genes of the SWI/SNF complex are also known to be associated with chromatin remodeling [26, 27, 28].

Considering the epigenetic roles of DNA methylation related to *IDH* mutation [6], chromatin remodeling associated with SWI/SNF alterations [8], and our findings of activated chromatin remodeling, histone modification, and DNA methylation pathways, alongside the rarity of genetic alterations, it appears that SNUC and similar undifferentiated carcinomas arising in the sinonasal tracts predominantly employ epigenetic mechanisms in their carcinogenesis.

Based on the spatial transcriptome profiles of SNUC tumor tissues, EZH2 appears to play a pivotal role in activating the PRC‐related chromatin remodeling process. This observation is highlighted by the fact that other PRC‐related genes, such as SUZ12, EED, and BMI1, did not exhibit notable prominence in the SNUC transcriptome. Concerning histone modification, it could either be a consequence of EZH2‐led PRC activation or occur concurrently. Notably, our spatial transcriptome analysis did not reveal direct activation of HDAC or HAT. Therefore, it seems plausible that EZH2 acts as an initiating factor, leading to subsequent signaling in chromatin remodeling and histone modification during the development of SNUC.

Epigenetic modifications possess the ability to control chromatin states and gene expression through processes such as DNA methylation and demethylation, histone modification, and chromatin remodeling, among others, all without altering the underlying DNA sequences [29, 30].

EZH2 is a member of the polycomb group proteins and serves as a key epigenetic regulator by functioning as a histone methyltransferase. It acts as the catalytic component within PRC2, responsible for the trimethylation of histone H3 at Lys 27 (H3K27me3). This methylation leads to gene silencing, impacting a wide array of biological functions such as cell cycle regulation, proliferation, and differentiation [31]. The role of EZH2 in cancer progression is increasingly recognized, with its heightened expression observed in various malignancies. EZH2 assumes an oncogenic role in a range of cancers, including solid tumors like breast cancer [32], prostate cancer [33], esophageal cancer [34], gastric cancer [35], anaplastic thyroid carcinoma [36], and endometrial carcinoma [37] as well as in hematologic malignancies [38] including follicular lymphoma [39]. In malignant tumor models, EZH2 is key in promoting tumor growth and metastasis [40, 41]. As previously mentioned, due to the numerous critical functions of EZH2 in cancer, therapeutic strategies targeting EZH2 have become essential in treating various types of cancer [41, 42].

The observed relationship between EZH2 and SNUC tumorigenesis indicates a significant role for EZH2 in the development of SNUC, positioning it as a potential target for EZH2‐focused therapies. Furthermore, by integrating our study findings with previously known characteristics of undifferentiated carcinoma in the sinonasal tract, we propose that EZH2‐activated SNUC represents a molecular subset of SNUC and its analogues.

In consideration of tumor immunity, distinct immune cell profiles were observed in the SNUC tissue microenvironment, notably naïve B cells, CD8 cytotoxic T cells, regulatory T cells (Tregs), and macrophages. These cells are recognized for their pivotal roles in modulating the tumor microenvironment, which can either promote antitumoral or protumoral responses [12, 13]. Furthermore, elevated expression of LAG3 was observed in tumor‐infiltrating immune cells. LAG3 (lymphocyte‐activation gene 3) is an immune checkpoint receptor primarily located on T cells. It functions as a coinhibitory receptor, modulating T cell activation and function. Furthermore, LAG3 has been found to be expressed on tumor‐infiltrating CD4^+^ and CD8^+^ T cells [43].

Epigenetic regulation, including EZH2, is recognized to play a central role in modulating T‐cell immune responses, particularly in the differentiation and recruitment of CD8^+^ T cells, CD4^+^ T cells, and Tregs in various types of malignancies. EZH2 is considered a major driver of immunomodulation and an immune escape regulator within the TIME [44, 45]. Although the plausible correlation between EZH2 and TIME in SNUC is not well understood, our findings may suggest the role of EZH2 in shaping distinct immune profiles in the SNUC microenvironment. Due to the lack of tissues, we could not investigate further the immune cell types in clinical samples. To explore any potential role of EZH2 in shaping a distinct TIME in SNUC, further studies using animal models that allow observation of the time‐series development of tumors may be warranted.

In conclusion, SNUC presents a significant challenge in oncology due to its rarity, aggressive nature, and limited treatment options. This study aimed to elucidate the molecular characteristics of SNUC, particularly focusing on tumorigenesis and tumor immunity. Our findings suggest that SNUC predominantly utilizes epigenetic mechanisms in tumorigenesis, favoring chromatin remodeling over genetic alterations. Notably, EZH2 activation was observed in SNUC tumor cells, indicating its crucial role in the carcinogenesis of SNUC. Therefore, targeting EZH2 with inhibitors may present a promising strategy for enhancing treatment outcomes. However, this study has its limitations, and further research is essential to fully understand the regulatory mechanisms behind EZH2 activation and its implications for therapy.

## Author Contributions

**Hyang Joo Ryu:** data curation (equal), funding acquisition (equal), methodology (equal), writing – original draft (equal). **Chung Lee:** formal analysis (supporting), methodology (supporting). **Sun Och Yoon:** conceptualization (equal), funding acquisition (equal), methodology (equal), project administration (equal), supervision (equal), writing – review and editing (equal).

## Disclosure

The authors declare no conflicts of interest and have all actively participated in the planning, execution, and analysis of this study, approving the final version submitted.

## Ethics Statement

This study received ethical approval from the Institutional Review Board (IRB) of Severance Hospital, Yonsei University Health System prior to commencement (IRB no. 4‐2022‐0466).

## Consent

The need for informed consent to participate was waived by the Institutional Review Board (IRB) at Institutional Review Board of Severance Hospital, Yonsei University Health System.

## Conflicts of Interest

The authors decalre no conflicts of interest.

## Supporting information


Figure S1.

**Table S1.** Comprehensive data from the Whole Transcriptome Atlas (Human RNA) utilizing multiplex digital spatial profiling (DSP) analysis for sinonasal undifferentiated carcinoma.

Table S2.

**Table S3.** Differential expression of genes in sinonasal undifferentiated carcinoma compared to normal epithelial cells within CK‐positive cell populations.

Table S4.

Table S5.

**Table S6.** Differential gene expression in tumor‐infiltrating immune cells versus immune cells in tumor‐free areas among CD45‐positive cell populations.

## Data Availability

All data generated and analyzed during this study are included in this article and its Supporting Information files.
