# Supplementary material for: Epigenetic and Immune Profile Characteristics in Sinonasal Undifferentiated Carcinoma
Source: Cancer Med. 2024 Nov 20;13(22):e70413. doi: 10.1002/cam4.70413 (PMC11577451; doi:10.1002/cam4.70413)
Supplement: Supplementary file 1 — Figure S1. [file CAM4-13-e70413-s002.pptx]

## Slide 1
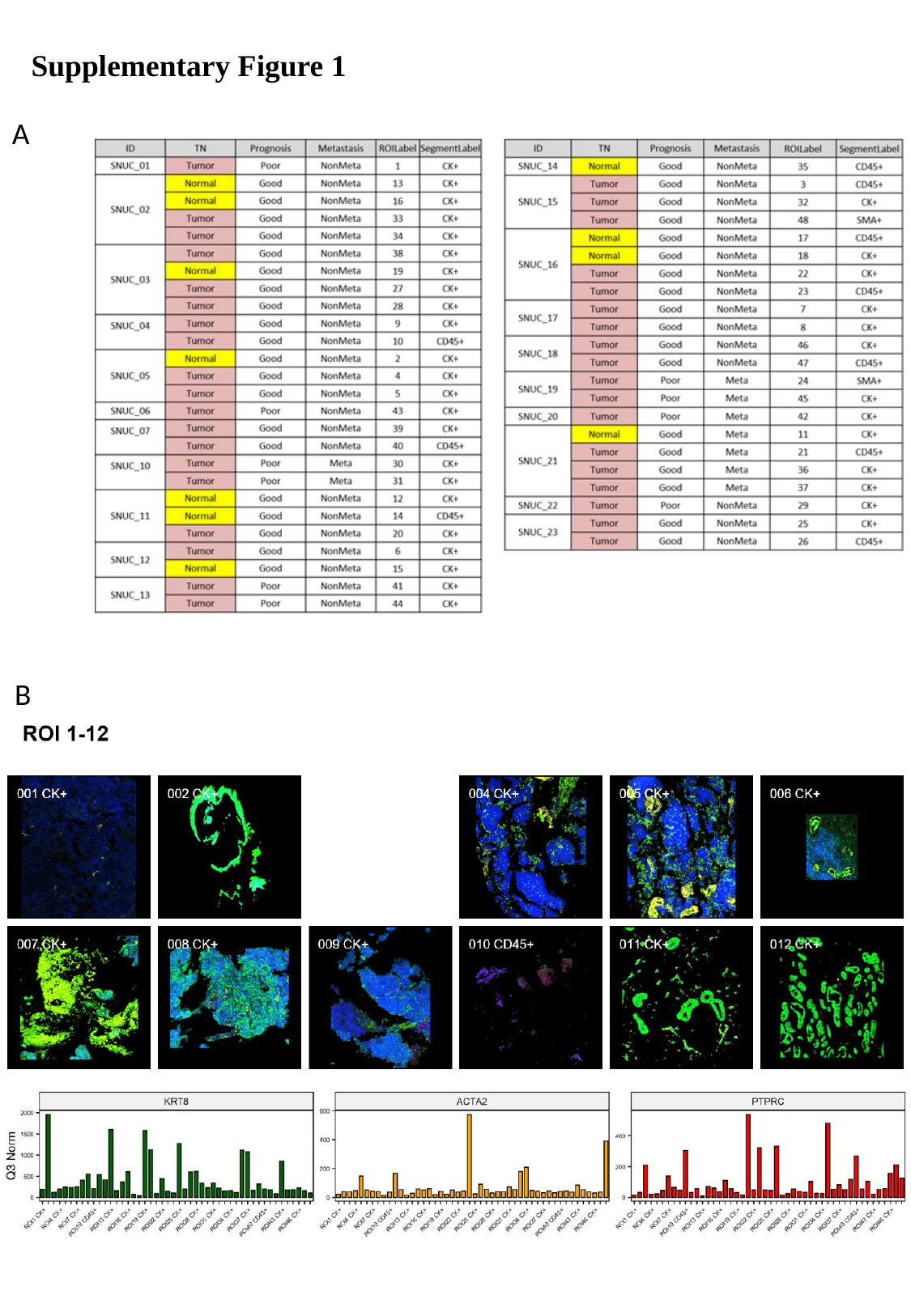

Supplementary Figure 1
A
B

## Slide 2
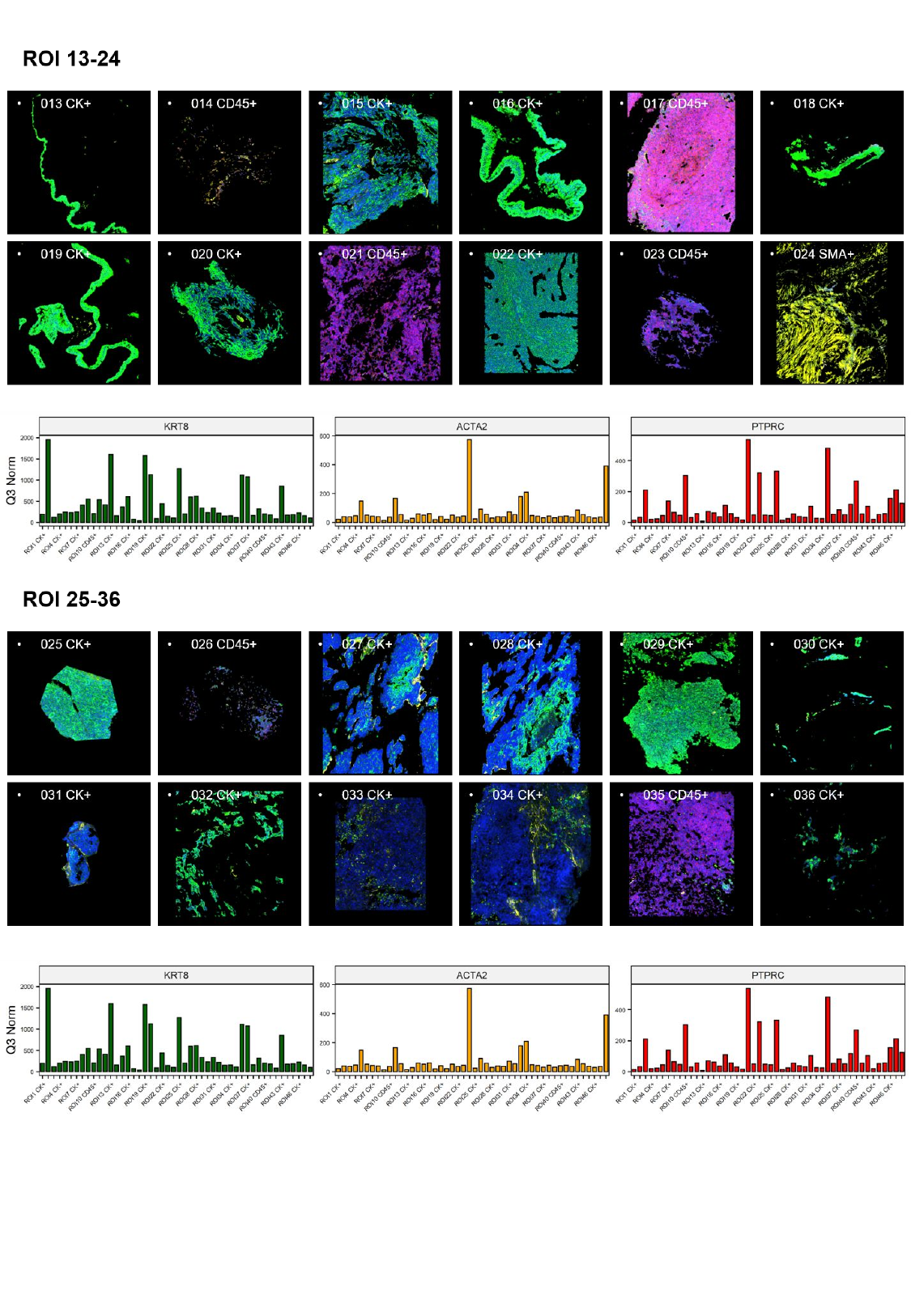

## Slide 3
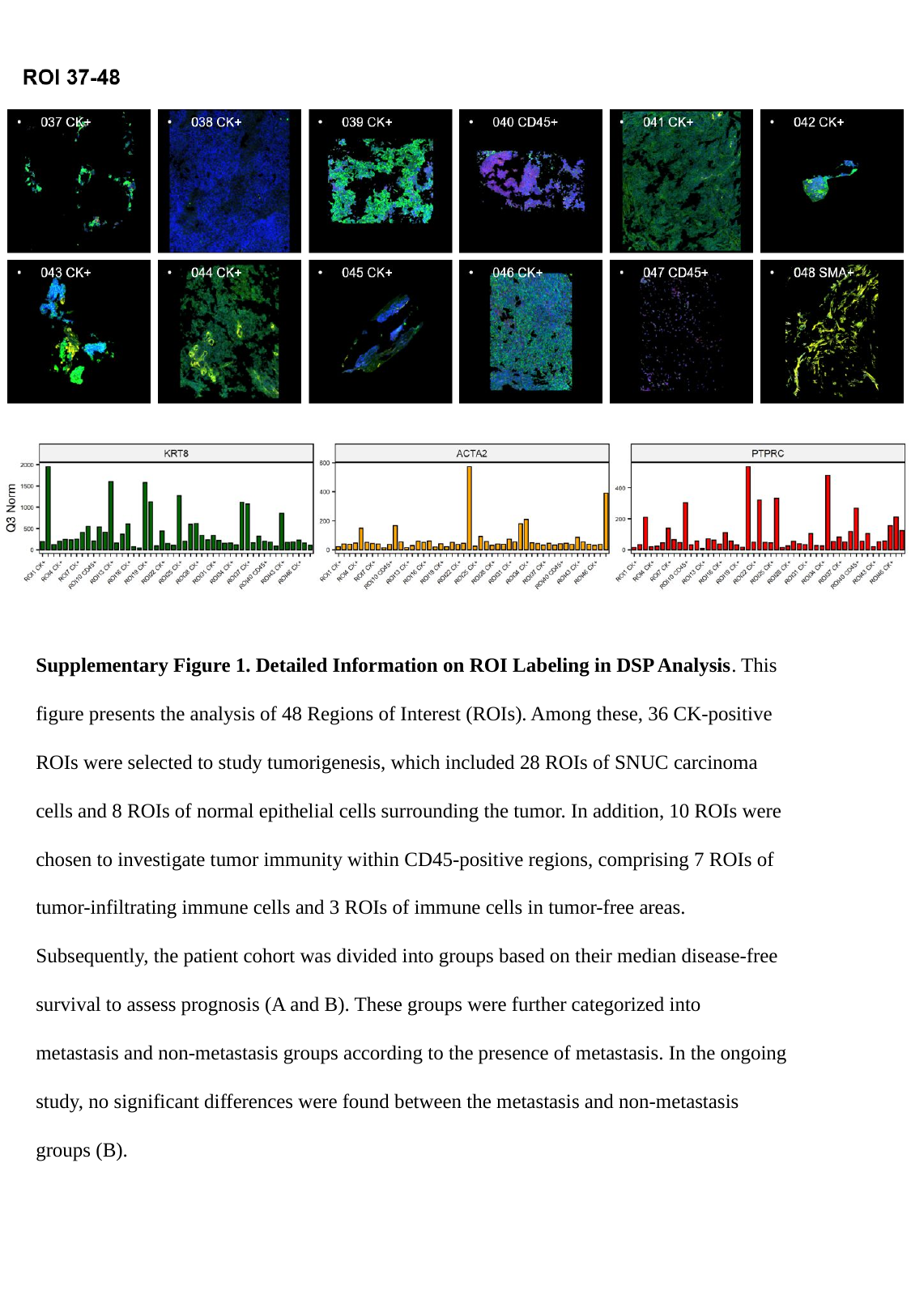

Supplementary Figure 1. Detailed Information on ROI Labeling in DSP Analysis. This figure presents the analysis of 48 Regions of Interest (ROIs). Among these, 36 CK-positive ROIs were selected to study tumorigenesis, which included 28 ROIs of SNUC carcinoma cells and 8 ROIs of normal epithelial cells surrounding the tumor. In addition, 10 ROIs were chosen to investigate tumor immunity within CD45-positive regions, comprising 7 ROIs of tumor-infiltrating immune cells and 3 ROIs of immune cells in tumor-free areas.
Subsequently, the patient cohort was divided into groups based on their median disease-free survival to assess prognosis (A and B). These groups were further categorized into metastasis and non-metastasis groups according to the presence of metastasis. In the ongoing study, no significant differences were found between the metastasis and non-metastasis groups (B).

## Slide 4
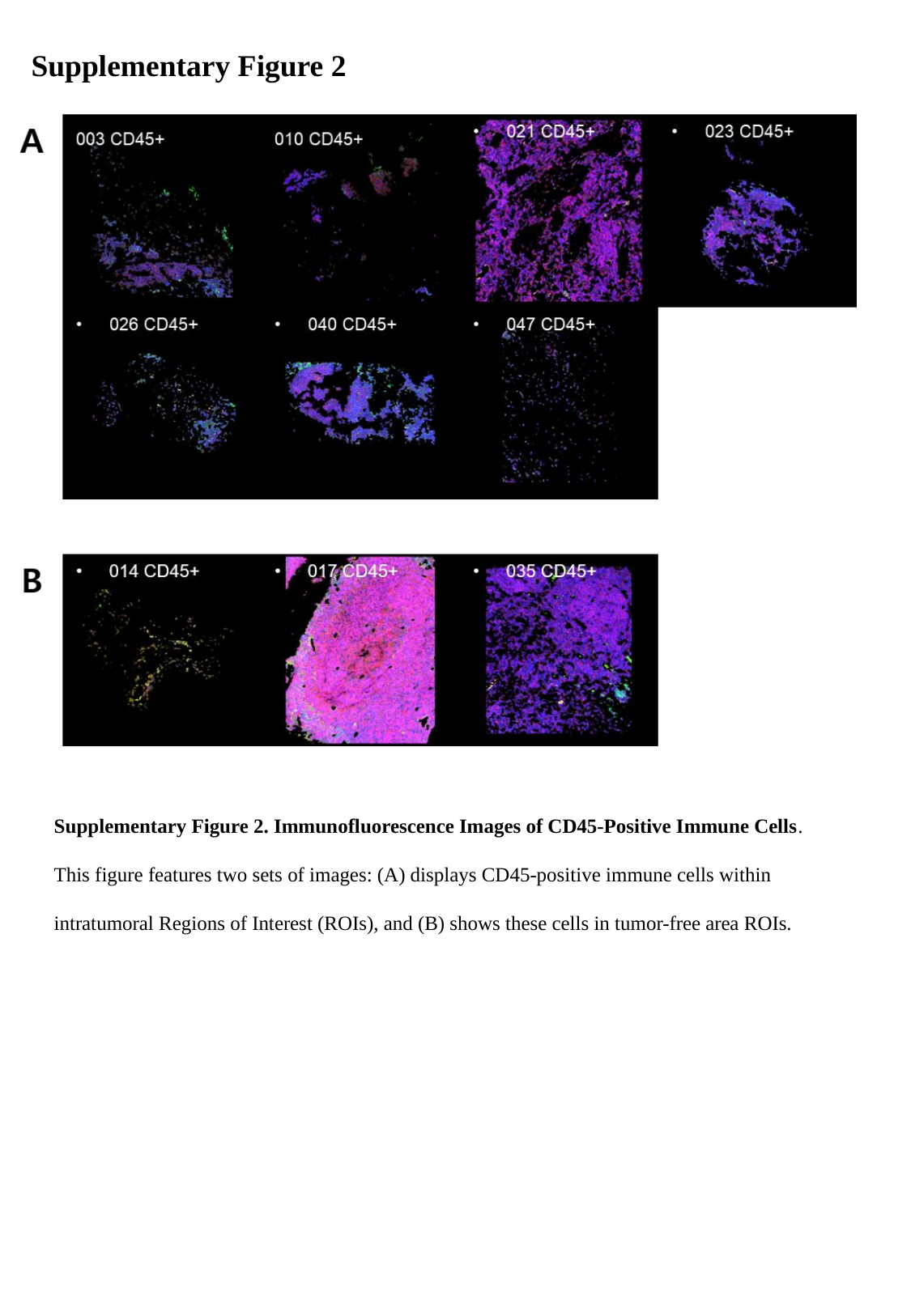

Supplementary Figure 2
Supplementary Figure 2. Immunofluorescence Images of CD45-Positive Immune Cells. This figure features two sets of images: (A) displays CD45-positive immune cells within intratumoral Regions of Interest (ROIs), and (B) shows these cells in tumor-free area ROIs.

## Slide 5
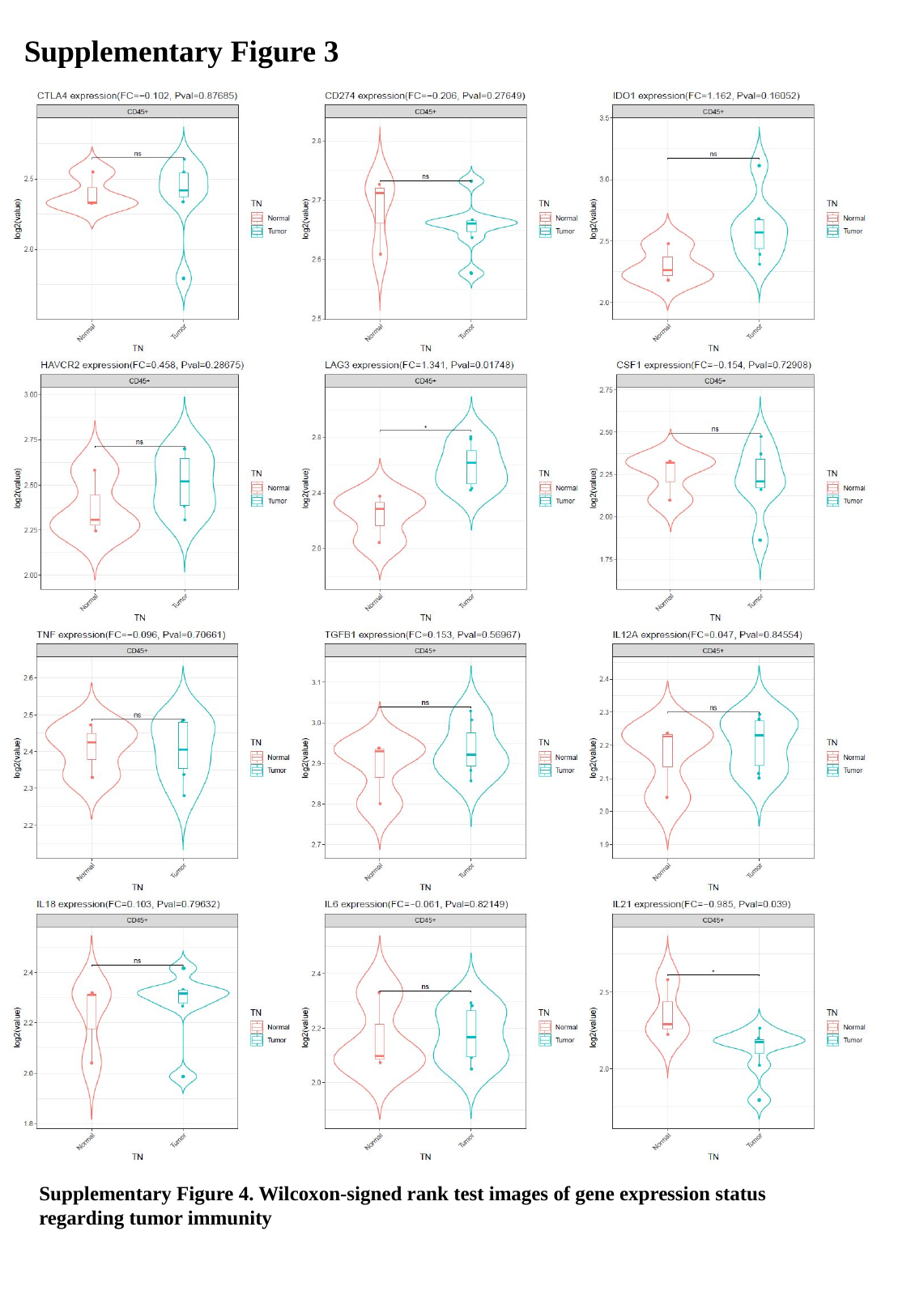

Supplementary Figure 3
Supplementary Figure 4. Wilcoxon-signed rank test images of gene expression status regarding tumor immunity
