# Supplementary material for: Epigenetic and Immune Profile Characteristics in Sinonasal Undifferentiated Carcinoma
Source: Cancer Med. 2024 Nov 20;13(22):e70413. doi: 10.1002/cam4.70413 (PMC11577451; doi:10.1002/cam4.70413)
Supplement: Supplementary file 5 — Table S4. [file CAM4-13-e70413-s007.docx]

**Supplementary Table 4. Brief summary of DEG analysis for genes upregulated in SNUC carcinoma cells**

| Gene | Fold change | Std..Error | t.value | P-val | Significance | GSEA pathway_  count  (TOP12) |
| --- | --- | --- | --- | --- | --- | --- |
| *H3C2* | 2.854888236 | 0.648595 | 4.401651 | 0.000101 | 3.995636 | 12 |
| *H3C7* | 2.232229628 | 0.530473 | 4.207999 | 0.000178 | 3.750315 | 12 |
| *H3C13* | 2.157442542 | 0.490556 | 4.397952 | 0.000102 | 3.990929 | 12 |
| *H3C15* | 2.104124362 | 0.508755 | 4.135834 | 0.000219 | 3.659526 | 12 |
| *H3C10* | 2.008093593 | 0.471222 | 4.261463 | 0.000152 | 3.817807 | 12 |
| *H2BC11* | 1.990715727 | 0.470389 | 4.232061 | 0.000166 | 3.780667 | 11 |
| *H2AC11* | 1.880055791 | 0.43 | 4.372227 | 0.00011 | 3.958214 | 5 |
| *H2BC9* | 1.87531293 | 0.518397 | 3.617522 | 0.000954 | 3.020283 | 11 |
| *H2AC12* | 1.843989603 | 0.453905 | 4.062498 | 0.000271 | 3.567655 | 5 |
| *H2BC10* | 1.839533645 | 0.4391 | 4.189327 | 0.000188 | 3.726789 | 11 |
| *H2AC17* | 1.754064515 | 0.450281 | 3.895489 | 0.000436 | 3.360055 | 5 |
| *H2AC21* | 1.638865153 | 0.388597 | 4.217393 | 0.000173 | 3.76216 | 5 |
| *H2BC13* | 1.586954299 | 0.282253 | 5.62245 | 2.67E-06 | 5.574163 | 11 |
| *EZH2* | 1.557840722 | 0.258618 | 6.023719 | 8.02E-07 | 6.095652 | 3 |

Abbreviations: DEG, differentially expressed genes; SNUC, sinonasal undifferentiated carcinoma; GSEA, gene set enrichment analysis
