# Supplementary material for: Epigenetic and Immune Profile Characteristics in Sinonasal Undifferentiated Carcinoma
Source: Cancer Med. 2024 Nov 20;13(22):e70413. doi: 10.1002/cam4.70413 (PMC11577451; doi:10.1002/cam4.70413)
Supplement: Supplementary file 6 — Table S5. [file CAM4-13-e70413-s004.docx]

**Supplementary Table 5. Brief summary of DEG analysis for genes downregulated in SNUC carcinoma cells**

| Gene | Fold change | Std..Error | t.value | Pval | Significance | GSEA pathway_  count  (TOP12) |
| --- | --- | --- | --- | --- | --- | --- |
| *BPIFA1* | -4.89333 | 0.78097 | -6.26572 | 3.90E-07 | 6.408961 | 1 |
| *BPIFB1* | -4.3636 | 0.685944 | -6.36145 | 2.93E-07 | 6.532544 | 1 |
| *LCN2* | -3.95908 | 0.590786 | -6.70138 | 1.07E-07 | 6.969271 | 1 |
| *FOS* | -2.83266 | 0.776643 | -3.64732 | 0.000878 | 3.056321 | 1 |
| *CLU* | -2.60435 | 0.593541 | -4.38782 | 0.000105 | 3.978042 | 1 |
| *MUC5B* | -2.54074 | 0.655163 | -3.87802 | 0.000459 | 3.338485 | 3 |
| *KRT7* | -2.50543 | 0.718719 | -3.48596 | 0.001373 | 2.86237 | 1 |
| *EGR1* | -2.17603 | 0.702242 | -3.09868 | 0.003886 | 2.410479 | 1 |
| *CP* | -2.15953 | 0.510782 | -4.22789 | 0.000168 | 3.775404 | 2 |
| *CYP4B1* | -2.14791 | 0.532214 | -4.0358 | 0.000292 | 3.534318 | 2 |
| *F3* | -2.1393 | 0.462318 | -4.62734 | 5.20E-05 | 4.284165 | 1 |
| *RRAD* | -2.13255 | 0.477049 | -4.4703 | 8.26E-05 | 4.083125 | 1 |
| *MUC4* | -2.01112 | 0.425844 | -4.72266 | 3.92E-05 | 4.406716 | 3 |
| *ALDH1A1* | -1.83849 | 0.609454 | -3.01661 | 0.004814 | 2.317518 | 2 |
| *HTN3* | -1.81424 | 0.821127 | -2.20945 | 0.03398 | 1.468776 | 1 |
| *SLC44A4* | -1.78751 | 0.386299 | -4.62728 | 5.20E-05 | 4.284083 | 2 |
| *MUC1* | -1.76002 | 0.4738 | -3.71469 | 0.000728 | 3.138159 | 3 |
| *CES1* | -1.67024 | 0.45108 | -3.70276 | 0.000752 | 3.123636 | 2 |
| *ID1* | -1.66439 | 0.570341 | -2.91824 | 0.006201 | 2.207537 | 1 |
| *MUC5AC* | -1.61959 | 0.481108 | -3.36639 | 0.001903 | 2.720671 | 3 |
| *ALDH3A1* | -1.60814 | 0.485718 | -3.31085 | 0.002211 | 2.655488 | 2 |

Abbreviations: DEG, differentially expressed genes; SNUC, sinonasal undifferentiated carcinoma; GSEA, gene set enrichment analysis
